# A new Activity Monitor for Aquatic Zooplankter (AMAZE) allows the recording of swimming activity in wild-caught Antarctic krill (*Euphausia superba*)

**DOI:** 10.1038/s41598-024-67999-3

**Published:** 2024-07-23

**Authors:** Lukas Hüppe, Dominik Bahlburg, Michael Busack, Johannes Lemburg, Laura Payton, Nils Reinhard, Dirk Rieger, Charlotte Helfrich-Förster, Bettina Meyer

**Affiliations:** 1 Neurobiology and Genetics, University of Würzburg, Biocentre, Theodor-Boveri-Institute, Am Hubland, 97074 Würzburg, Germany; 2 Section Polar Biological Oceanography, Alfred Wegener Institute, Helmholtz Centre for Polar and Marine Research, Am Handelshafen 12, 27570 Bremerhaven, Germany; 3 Section Deep-Sea Ecology and Technology, Alfred Wegener Institute, Helmholtz Centre for Polar and Marine Research, Am Handelshafen 12, 27570 Bremerhaven, Germany; 4 Scientific Workshop, Alfred Wegener Institute, Helmholtz Centre for Polar and Marine Research, Am Handelshafen 12, 27570 Bremerhaven, Germany; 5 CNRS, INP, EPOC, UMR 5805, University of Bordeaux, 33120 Arcachon, Bordeaux France; 6 Institute for Chemistry and Biology of the Marine Environment, University of Oldenburg, Carl-Von-Ossietzky-Straße 9-11, 26111 Oldenburg, Germany; 7 Helmholtz Institute for Functional Marine Biodiversity at the University of Oldenburg (HIFMB), Ammerländer Heerstrasse 231, 26129 Oldenburg, Germany

**Keywords:** Antarctic Krill, *Euphausia superba*, Swimming behavior, Locomotor activity, Diel vertical migration (DVM), Ecology, Behavioural ecology

## Abstract

Antarctic krill (*Euphausia superba*, hereafter krill) is a pelagic living crustacean and a key species in the Southern Ocean ecosystem. Krill builds up a huge biomass and its synchronized behavioral patterns, such as diel vertical migration (DVM), substantially impact ecosystem structure and carbon sequestration. However, the mechanistic basis of krill DVM is unknown and previous studies of krill behavior in the laboratory were challenged by complex behavior and large variability. Using a new experimental set-up, we recorded the swimming activity of individual wild-caught krill under light–dark cycles. Krill individuals exhibited differential phototactic responses to the light regime provided. However, using a new activity metric, we showed for the first time a consistent nocturnal increase in krill swimming activity in a controlled environment. Krill swimming activity in the new set-up was strongly synchronized with the light–dark cycle, similar to the diel vertical migration pattern of krill in the field when the krill were sampled for the experiment, demonstrated by hydroacoustic recordings. The new set-up presents a promising tool for investigating the mechanisms underlying krill behavioral patterns, which will increase our understanding of ecological interactions, the spatial distribution of populations, and their effects on biogeochemical cycles in the future.

## Introduction

Antarctic krill (*Euphausia superba*, hereafter krill) is an up to 6 cm long crustacean, endemic to the Southern Ocean. With an estimated biomass of 300 – 500 million tons^1^, it is one of the biomass richest species on earth^2^. By grazing mainly on phytoplankton and being the major prey for iconic predators, such as whales, seals, penguins, and flying seabirds^3^, krill takes a key position in the Southern Ocean ecosystem, linking energy from the base of the food web into the highest trophic levels. The Southern Ocean is a considerable carbon sink that accounts for about 40% of the global ocean uptake of anthropogenic CO_2_^4^. With their large biomass, krill contribute substantially to carbon transport from the surface to the deep ocean^5,6^.

One means of carbon transport to the deep is by diel vertical migration (DVM) of zooplankton^7^, a behavior commonly characterized by an ascent at night to feed on phytoplankton in the upper water column and a descent to deeper and darker waters during the day to hide from visually hunting predators^8^. DVM is a widespread phenomenon performed by zooplankton organisms in all world oceans, making it the biggest migration in terms of biomass on Earth^9^. Besides its contribution to carbon sequestration, the synchronized movements of such a large biomass actively shape predator–prey interactions^10^. In addition, the vertical movement in the water column could also impact the horizontal distribution patterns of organisms, as currents can differ substantially in direction and velocity between the surface and deeper waters^11,12^. Although “regular” nocturnal DVM behavior, with upwards migration during the night, has been commonly observed in Antarctic krill^13–15^, there is considerable variation, including reverse DVM (i.e. upwards migration during the day), higher frequency migrations or no synchronized migrations^15–17^. While the ultimate factors driving zooplankton diel vertical migration (DVM) are widely recognized, the precise mechanisms governing the daily manifestation of this behavioral pattern remain elusive^18^.

Light intensity is considered the most important proximate cue for DVM^9,19^, with animals following isolumes (i.e. depths of constant light intensity;^20^) and responding to intensity changes caused by cloud cover^21^ or lunar illumination^22^. But also, endogenous clocks have been shown to control vertical migration in marine zooplankton^19,23,24^, including krill^25,26^. Other internal and external factors may further modulate DVM^15,19^, which can lead to the great variability in observed patterns^15,16^.

Most studies on krill DVM are based on in-situ observations, using depth-stratified net catches or hydroacoustic observations. Only a few studies focus on investigating mechanistic principles under controlled laboratory conditions^25,26^. Understanding the mechanistic principles behind behavioral patterns of pelagic key species, such as krill, is important to understand ecological interactions, the spatial distribution of populations, and their effects on biogeochemical cycles, especially in relation to carbon sequestration^27,28^.

Investigations into the mechanisms underlying animal behavior require the study of behavioral output under controlled conditions. Previous approaches used video recordings of a group of krill in a vertical tank set-up in the laboratory to record diel behavioral patterns^25^, another study used an activity monitor, which recorded the beam breaks of infrared light barriers at the bottom and top of vertical tubes to observe krill vertical migration behavior^26^. While these pioneering studies gave first insights into the drivers of krill swimming behavior, complex behavior, and unnatural behavioral phenotypes prevented more detailed insights into the mechanisms underlying krill behavior.

In terrestrial model organisms, such as the fruit fly (*Drosophila melanogaster*) or mouse (*Mus musculus*), locomotor activity is commonly used as a behavioral read-out to study how internal and external factors influence behavior and has enabled a detailed characterization of the mechanisms underlying behavioral activity^29,30^. Several experimental set-ups were developed to record behavioral activity in model organisms, and some are commercially available (e.g. Drosophila Activity Monitor, Trikinetics Inc.;^31^). In contrast, experimental work with polar marine organisms, such as krill, poses a multitude of challenges, such as the maintenance of krill in the laboratory at low temperatures for extended periods of time, expensive logistics, competitive ship time, and difficult access to polar regions, especially during winter.

To overcome some of these challenges, we used previous experiences from krill behavior studies^25,26^ to develop and build a new independent and transportable Activity Monitor for Aquatic Zooplankter (AMAZE). Here, we present data from a first experiment, where we installed the set-up on a commercial krill fishing vessel and recorded krill swimming behavior of wild-caught krill for several days under light–dark (LD) cycles. We compare the findings on krill activity with hydroacoustic observations of swarm behavior in the field and discuss the potential use of this set-up in future studies on swimming behavior of krill and other pelagic species.

## Material and methods

### Activity monitor for aquatic zooplankter (AMAZE)

**Figure 1:**
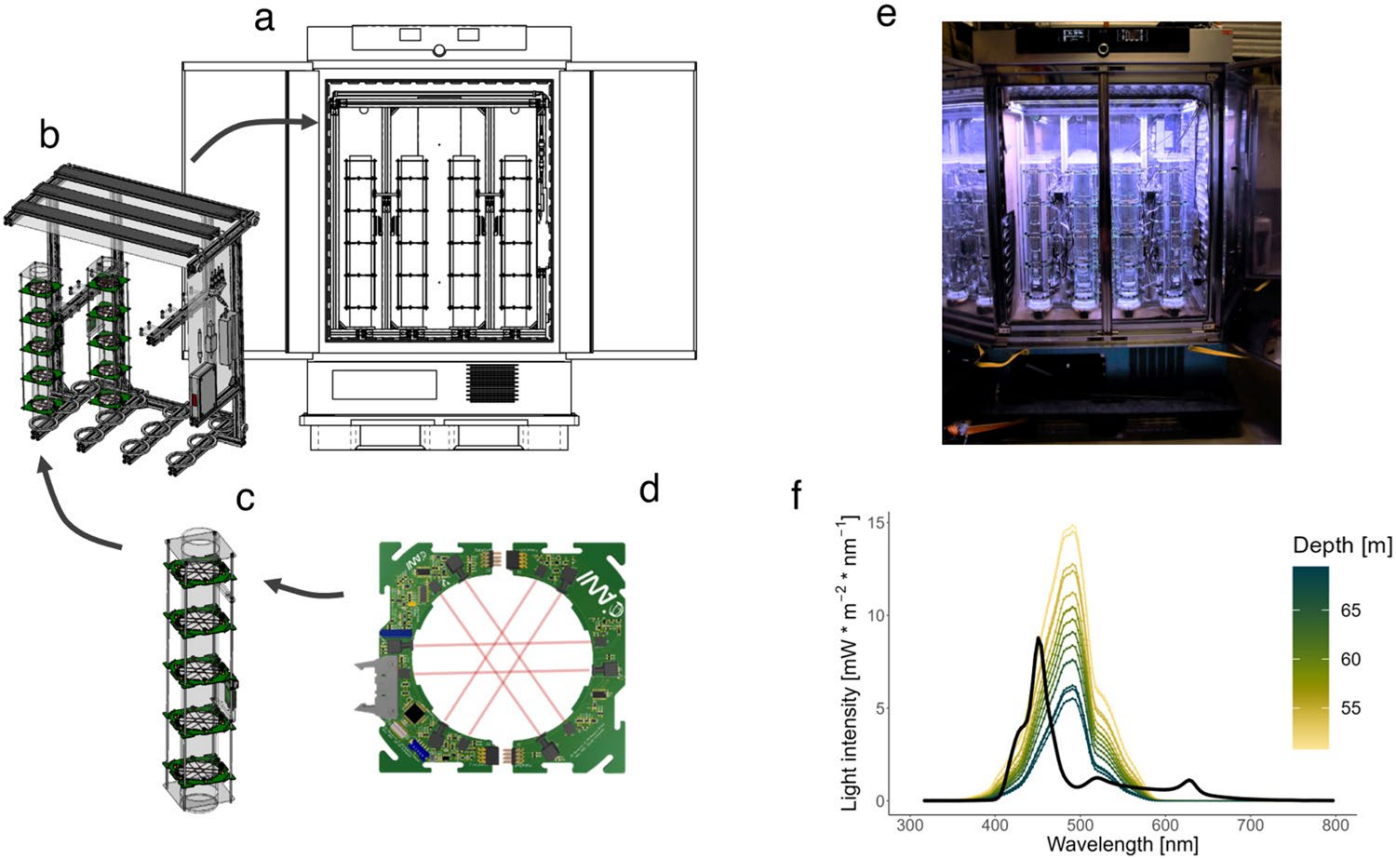
Description of the AMAZE set-up. (**a**) The incubator housing the activity monitor set-up for precise temperature control during the experiments. (**b**) The structure that provides the mounting spaces for the experimental columns, programmable LED light bars, power supply, and data transfer. (**c**) Experimental column (height: 80 cm, diameter: 9 cm) equipped with infrared light-barrier system. (**d**) Custom-designed detector module, consisting of two circuit boards fitted with six pairs of IR light barriers. (**e**) The AMAZE set-up installed on the krill fishing vessel Antarctic Endurance. (**f**) Comparison of midday light spectrum in the activity monitor (black line) and in the field between 50 and 70 m water depth (colored lines).

The basis of the activity monitor is a compressor-cooled incubator (ICP750eco, Memmert GmbH + Co. KG, Germany; Fig. 1a) with precise programmable temperature regulation (− 12°C to 60°C, accuracy: 0.1°C), which allows simulating a wide range of temperature habitats (i.e. tropical to polar water temperatures). The incubator is mounted onto a plastic transport pallet, enabling transport and installation in challenging environments, such as research and fishing vessels and field stations. The footprint of the whole set-up totals 130 × 110 cm. The incubator houses a removable aluminum frame (Fig. 1b) that provides 12 mounting spaces for experimental columns, a structure for mounting a lighting system, and the components to power and control all electronic parts.

To approximate the light spectrum and intensity of various underwater light environments and their daily dynamics, the set-up is equipped with three programmable LED light bars (Mitras Lightbar 2 Actinic, GHL Advanced Technology GmbH & Co. KG, Germany), mounted on top of the aluminum frame. The intensity of the seven color channels of the light bars can be controlled individually, which allows the adjustment of the light spectrum and intensity to resemble natural underwater light fields in different habitats and the simulation of day-night cycles. The light bars are connected to a light computer (Profilux 4, GHL Advanced Technology GmbH & Co. KG) outside the incubator, which controls the light bars. A white acrylic sheet (white-diaphanous (WH73), Modulor GmbH, Germany) is placed beneath the LED bars to diffuse the light and to create a homogenous light field in the experimental chamber. Four fans (Sunon, Taiwan) at the bottom of the incubator aid a permanent air circulation in the incubator chamber to ensure a homogenous temperature distribution. Cardboard sheets at the bottom and the sides shield the experimental columns from the direct influence of the cooling elements of the incubator and further, ensure homogenous temperature distribution across all columns.

To record the activity of individual krill, up to 12 transparent acrylic glass columns (Fig. 1c) can be installed in the aluminum frame and allow the recording of up to 12 individual krill in parallel. During our experiment, we removed 2 columns from the set-up, for better air circulation, which aids a homogeneous temperature distribution when using the system at very low temperatures. The experimental columns, with a height of 80 cm and an inner diameter of 9 cm, are closed at the bottom and are filled with seawater up to 5 cm below the top of the column (water volume: ~ 6L, water column height: 75 cm).

The recording of individual behavioral activity is inspired by traditional locomotor activity monitoring systems based on the principle of animals breaking infrared (IR) light beams when moving^26,31,32^. For this, each column is equipped with five detector modules (Fig. 1d), equally spaced throughout the height of the column. The uppermost detector is placed 3 cm from the water surface so that animals swimming close to the surface regularly trigger the upper detector, and the lower detector is placed 5 cm from the bottom of the column so that dead animals will not trigger a detector at all. Each detector module comprises a custom-designed printed circuit board (PCB) comprising two complementary parts that plug together around the column. Each module has six pairs of IR LED emitters (940 nm) and receivers. The six IR light beams crossing the column are arranged in a way that minimizes the size of the gaps between the beams (max. gap size 32.4 mm; suppl. Figure 1). The IR emitters and receivers are equipped with 3D-printed apertures to focus the emitted IR light beam in the direction of the matching receiver and to shield the receivers from scattered light of other IR beams in the set-up. To further minimize interference between IR beams, only one beam is active at a time. Each beam is activated for 10 ms, resulting in approximately 16 rotations per second. A detection is only counted if the interruption lasts at least 50% of the beam activity time (i.e. 5 ms). Each detector module is equipped with a status and a detection LED, which provide information about the system's status during installation. A potentiometer on the master module allows manual adjustment of the intensity of the IR beams, which helps adjust the beam intensity to a level where translucent organisms, such as krill, can cause a beam break.

The power supply to and data acquisition from the detector modules is done via a single-board Raspberry Pi computer (Raspberry Pi 3 Model B, Raspberry Pi Foundation, UK; RPi) mounted to a panel on the side of each column. Its functionality is extended by a custom-designed add-on board (HAT; hardware attached on top), which carries a real-time clock (RTC), sensors for temperature, light, and humidity, a push button to control the status and detector LEDs on the detector modules and a power in- and outlet.

Data acquisition for each column is done via the RPi, which runs a Python script to check the status of the receivers on each detector module. Detections of each detector module are registered in a logfile on the RPi, along with a timestamp and a set of metadata, such as temperature, light, and humidity from the sensors on the RPi HAT. The RPis are connected to a network switch that can be accessed outside the set-up. Like this, the logfiles can be accessed at any time during the experiment to check for proper functioning of the system.

### Krill collection and behavior recordings

Antarctic krill (*Euphausia superba*) were sampled on February 16^th^, 2022 at 15:50 (local time, UTC-2) north of the South Orkney Islands (60.04°S, 46.45° W) using the continuous fishing system of the krill fishing vessel *Antarctic Endurance*. The vessel trawled at a speed of 1.5 – 2 knots using a commercial trawl at a depth of 94 m, and krill was pumped onboard by a vacuum hose connected to the cod-end of the trawl. On board, krill was separated from water on a metal grate, from which it was sampled. Sampled krill were kept in surface seawater at densities of ~ 1 Ind. per L, at 1 °C under constant darkness for an acclimation period of about 6 h, to reduce the impact of sampling stress and to check for the condition of krill individuals before transfer to the experimental set-up. The experimental columns were filled with filtered surface seawater, and columns were distributed to the set-up to cool down before the start of the experiment. The temperature during the experiment was set to 0.8°C, resembling field conditions.

At the start of the experiment, unharmed, adult krill of mixed sex (5 male, 5 female) were selected and individually distributed to the experimental columns. Subsequently, the beam break activity of 10 individual krill was recorded for 5 full days under light–dark cycles. Krill were not fed during the experiment. The light conditions in the activity monitor were set to approximate the light spectrum and intensity in the field at the time of sampling, with a photoperiod of 15.5 h and light intensities increasing and decreasing linearly from a maximum of 8.8 mW * m^-2^ during midday, which resembles midday light intensities at ~ 60 m depth during late Summer in the same region (Fig. 1f).

At the end of the experiment, krill were removed from the set-up and checked for overall condition. Total length was measured from the front of the eye to the tip of the telson, excluding setae, and sex was determined under a stereo microscope. No mortality was observed during the experiment.

### Behavioral data analysis

After an initial check of the data for technical errors, one of the 10 recorded individuals was removed due to technical errors in a detector module, leaving 9 individuals (4 male, 5 female) remaining for further analysis.

Raw beam break recordings were used to calculate beam break events for each detector module. A beam break event was defined as the detection of at least one beam break detection within a one-minute interval. These events were then summed into 10-min intervals, and the data were normalized by rescaling the values between 0 and 1 for each detector module. To enhance visualization, we applied a centered moving average over six of the 10-min bins.

To investigate daily rhythmicity underlying krill behavioral activity, we calculated a swimming activity metric by considering only the upward swimming movements of krill. To achieve this, we arranged the raw beam break data of all five detector modules of each experimental column in chronological order and only selected beam break detections that followed a detection at the detector module positioned lower on the column. The beam breaks caused by upward swimming were summed into 10-min intervals and normalized by rescaling the values between 0 and 1 for each column. Activity data were smoothed by a centered moving average applied over six values. It should be noted that upward swimming behavior as a swimming activity metric might not reflect other types of behavioral responses (e.g. horizontal movements, changes in swimming speed) to light–dark cycles.

To investigate behavioral differences between light phases (i.e. lights-on/lights-off), we used the non-parametric Mann–Whitney-U-Test (R *stats* package version 4.1.2) at a significance level of p < 0.05, for both beam break events and krill activity data. For average day analysis, we calculated the mean and standard error of the mean (s.e.m.) per 10-min time interval throughout the day for all individuals in one experiment. Data handling, analysis, and visualization was done in R (version 4.1.2;^33^), via RStudio (version 2023.12.1.402) using the *tidyverse* package (version 2.0.0^34^).

### Comparative light measurements in the field and the activity monitor

To compare the ambient light spectrum and intensity with the light settings in the activity monitor, we measured a vertical profile of spectral light intensity of the upper water column (< 200 m) using a RAMSES ACC G2 hyperspectral radiometer (TriOS GmbH, Germany). The sensor measures light intensity over a spectral range from 320 to 950 nm, along with temperature and pressure. The measurement was done on February 27^th^, 2022 within 30 min of local solar noon, north of the South Orkney Islands (60.06° S, 45.61° W). Light spectrum and intensity in the activity monitor were measured with the same sensor, placed in the center of the set-up, at the height of the top of the experimental columns, with the light spectrum and intensity set to the daily maximum during the LD experiment. Raw data were calibrated according to the measurement environment (i.e. underwater and atmospheric measurements, respectively) following the manufacturer’s instruction and software (msda_xe version 8.9.2). Pressure values of calibrated data from field measurements were converted to depth values, following the conversion formula for seawater applications used for standard oceanographic equipment^35^. For visualization purposes, the data were limited to a depth range between 50 and 70 m, where light intensity values matched the values measured in the activity monitor. Data handling and visualization were done with R, using the packages *tidyverse* and *scico* (version 1.3.1^36^).

### Hydroacoustic data recording

Hydroacoustic data were recorded from a hull-mounted ES80 echosounder (Kongsberg Maritime AS) aboard the Antarctic Endurance around the time of krill sampling. The signal received from the 200 kHz band was used to visualize the vertical distribution of krill swarms below the ship. As only data is used from periods of active fishing and the vessel is specifically targeting *E. superba*, it is highly probable that the recorded signal represents *E. superba* swarms. The raw acoustic data were converted to mean volume backscattering strength and binned to a time resolution of 1 s and depth bins of 0.5 m using Echopype^37^.

## Results

### A new transportable, independent activity monitor to record individual swimming activity in wild-caught Antarctic krill

The temperature- and light-controlled activity monitor AMAZE (Fig. 1a-e) is independent of temperature-controlled rooms. It only requires a standard power supply, making it flexible for use in various environments, such as research stations and ships. The behavioral activity of individual krill is recorded based on the principle of animals breaking infrared light barriers positioned at five different levels while swimming in a vertical experimental column (water column height: 75 cm, diameter: 9 cm; Fig. 1c,d). The recorded beam breaks provide information about the activity and approximate position of the krill in the experimental column at any given time. Controllable LED light bars at the top of the frame allow for the approximation of underwater light spectra and intensities, as shown by comparing the light intensity and spectrum in our activity monitor and the water column during our fieldwork. Although the peak spectral intensity in the activity monitor is slightly shifted (~ 450 nm) compared to the field measurements (~ 490nm), the light set-up can approximate the light spectrum and intensity that occur during midday in late summer in the upper water column (Fig. 1e). To ensure precise temperature control during experiments, we placed the aluminum frame inside a transportable incubator (Fig. 1a). This allows the simulation of a wide range of temperature regimes, including the low water temperatures in polar regions.

### Rhythmic nocturnal swimming activity is masked by positive phototaxis under simulated light–dark cycles

To investigate the behavioral activity of krill in the activity monitor, we sampled krill from the field and recorded their swimming behavior for 5 days under light–dark cycles, simulating the natural photoperiod and light conditions in the water column at the time of sampling. The simulated LD cycle was centered around local solar noon, and the light intensity increased and decreased linearly from lights-on to midday and from midday to lights-off, respectively.

**Figure 2:**
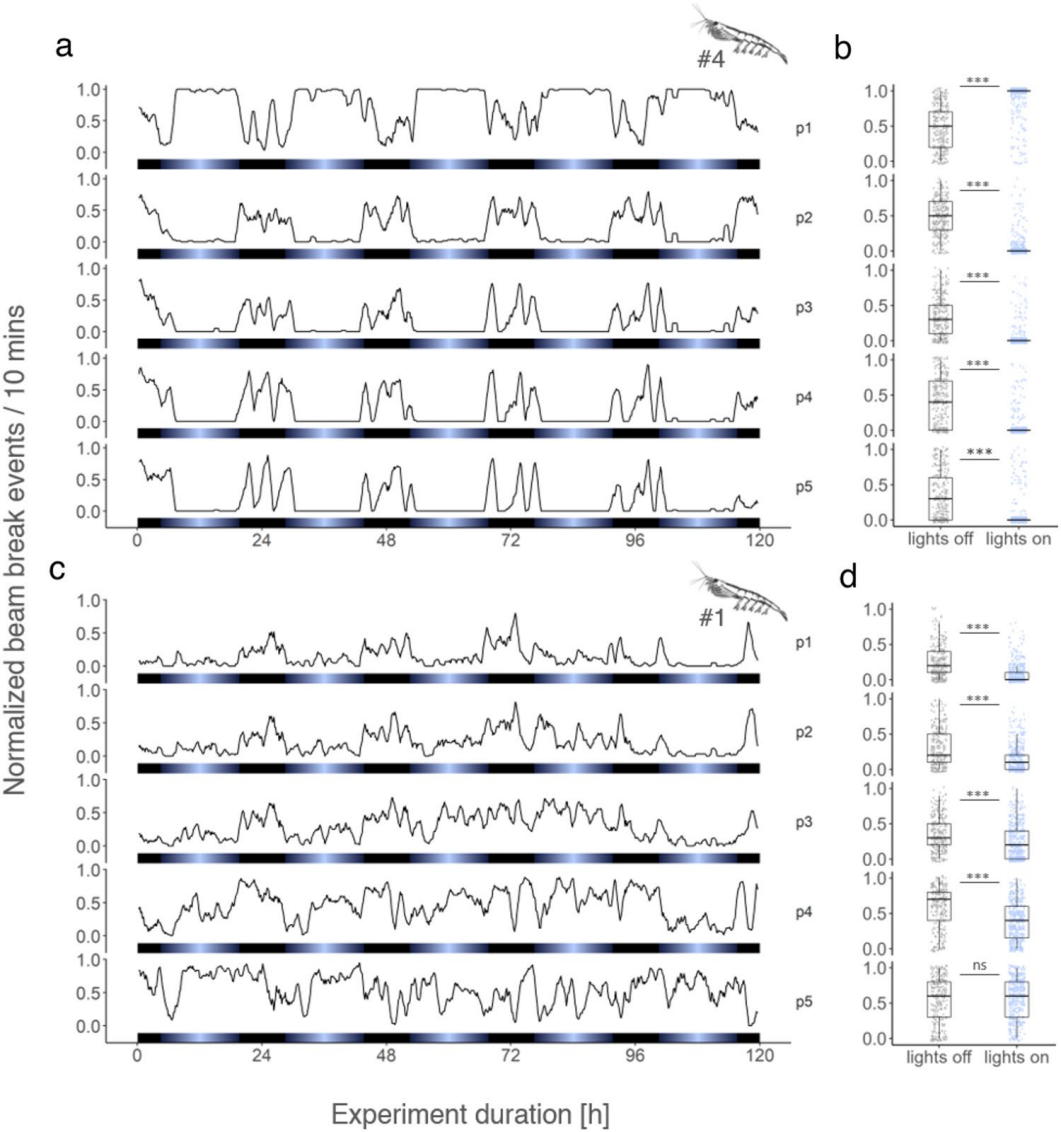
Swimming behavior of individual krill under light–dark cycles. Normalized beam break events of krill individuals #4 (**a**) and #1 (**c**) across the five detector modules (p1-p5) over the duration of the experiment. Corresponding box plots show normalized beam break events during lights-off and lights-on for both individuals at the five detector levels (**b**, **d**). Differences between light conditions have been tested with the Mann–Whitney-U-Test, using the following significance levels: p < 0.05: *, p < 0.01: **, p > 0.001: ***, p > 0.05: ns. Bars at the bottom of each plot depict the light regime.

**Figure 3:**
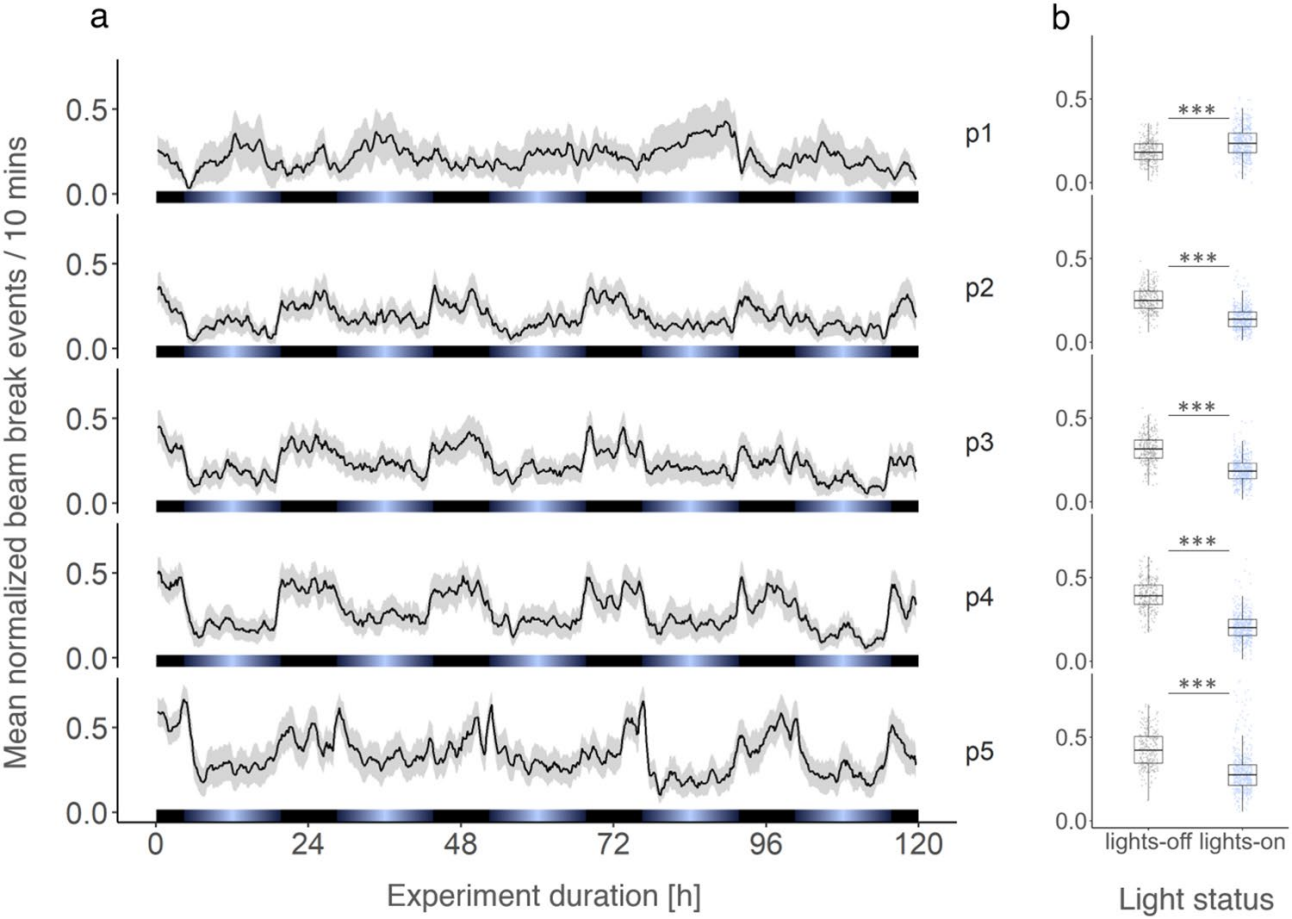
Group swimming behavior of krill under light–dark cycles. Group mean (n = 9) of normalized beam breaks across the five detector modules over the duration of the experiment (**a**). Lines with shading depict the mean ± standard error of the mean (s.e.m.), respectively. Box plots show group mean (n = 9) of normalized beam breaks during lights off and lights on for the five detector levels, respectively (**b**). Light bars and significance levels as in Fig. 2.

The data of the summed beam break events show krill with rhythmic swimming behavior synchronized to the provided light–dark cycle (Fig. 2a,c), showcasing the activity monitor´s ability to reflect rhythmic swimming activity in krill. The two examples of krill behavior in the activity monitor further showcase differences in phototactic response at the individual level, where individual #4 spends most of the time in the upper part of the column during the light phase (Fig. 2a,b). In contrast, individual #1 tends to stay in the lower part of the column during lights-on. However, irrespective of the individual’s response to the light source, ongoing swimming activity throughout the whole column during the dark phase can be observed in most individuals (Fig. 2 and suppl. Figure 2), suggesting that the attraction to the artificial light is masking nocturnal swimming behavior in individuals with a clear phototactic response. The individual variability in krill behavior especially towards the provided light is further reflected in the group behavior of all krill during the experiment (Fig. 3a). At the top detector position (p1), the day-night differences in mean beam break events of all krill show a tendency of being higher in the column during the light phase (Fig. 3b). In contrast, all other detector positions (i.e. p2 to p5) indicate higher group level activity during the dark phase. In summary, the analysis of krill activity data under light–dark cycles reveals complex behavioral patterns at the individual level. However, the data indicate a consistent trend of increased activity during the dark phase in most parts of the experimental column, at both individual and group levels.

### Disentangling aspects of swimming behavior reveals rhythmic, nocturnal swimming activity in krill

Krill are negatively buoyant, which means they must swim continuously to maintain their position in the water column^38^. When studying daily differences in krill swimming activity in spatially confined laboratory environments, it is a major challenge to distinguish between periods of *baseline swimming activity* and periods of *increased swimming activity*. In the activity monitor, a krill can trigger beam breaks by actively swimming upwards, actively or passively swimming downwards, or hovering (i.e. maintaining its position) at the level of a detector module. As our activity monitor allows us to spatially track an individual over the entire column, we used this data to separate the baseline from the increased swimming activity. To do this, we restricted the data to the upward swimming component as a conservative estimate of *swimming activity*.

**Figure 4:**
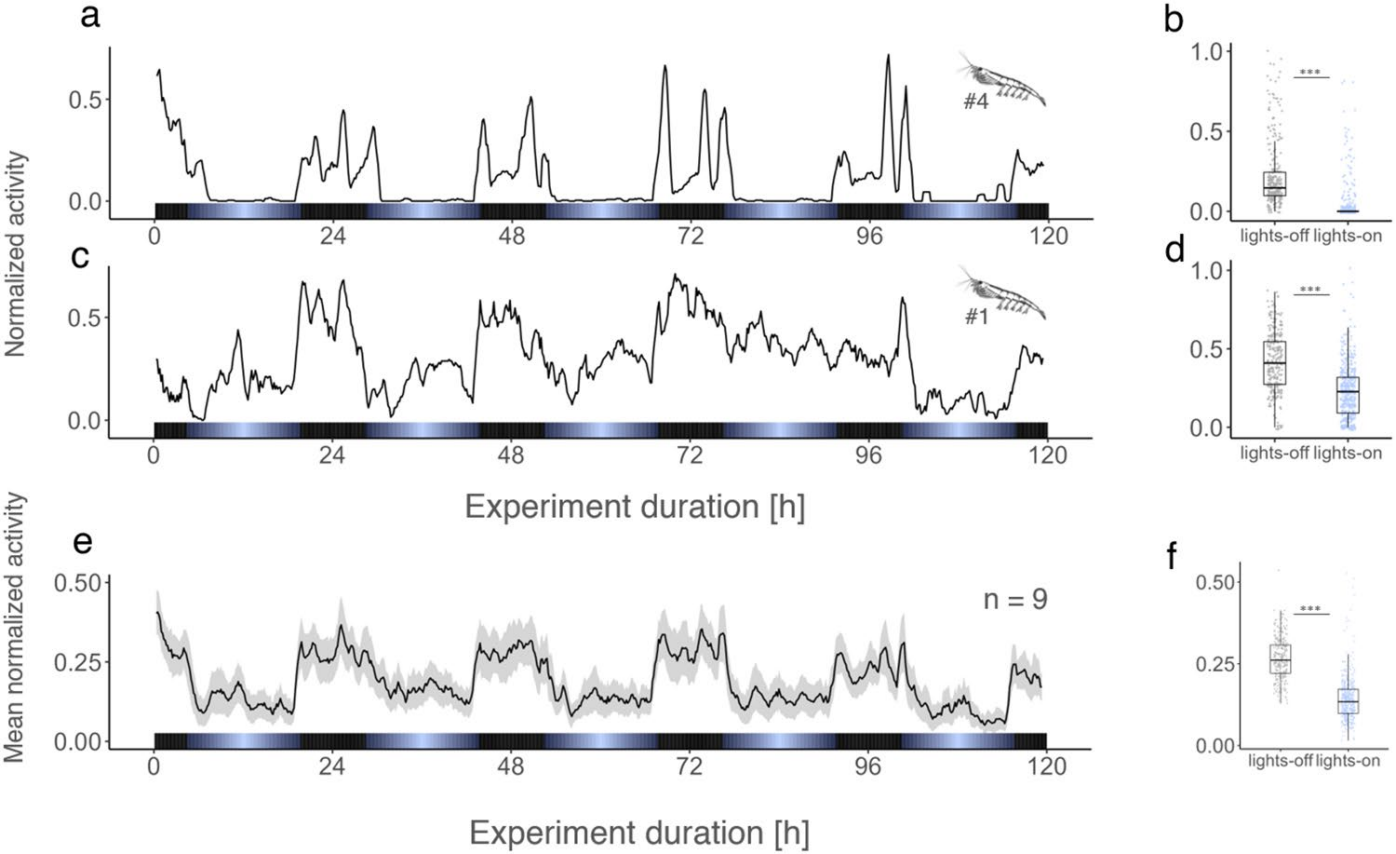
Individual and group mean swimming activity of krill under light–dark cycles. Normalized swimming activity of krill individuals #4 (**a**) and #1 (**c**), and group swimming activity (group mean ± s.e.m., n = 9) (e) under light–dark cycles over the duration of the experiment. Box plots show individual (**b**, **d**) and group mean (**f**) of normalized swimming activity during lights-off and lights-on phases. Light bars and significance levels as in Fig. 2.

**Figure 5:**
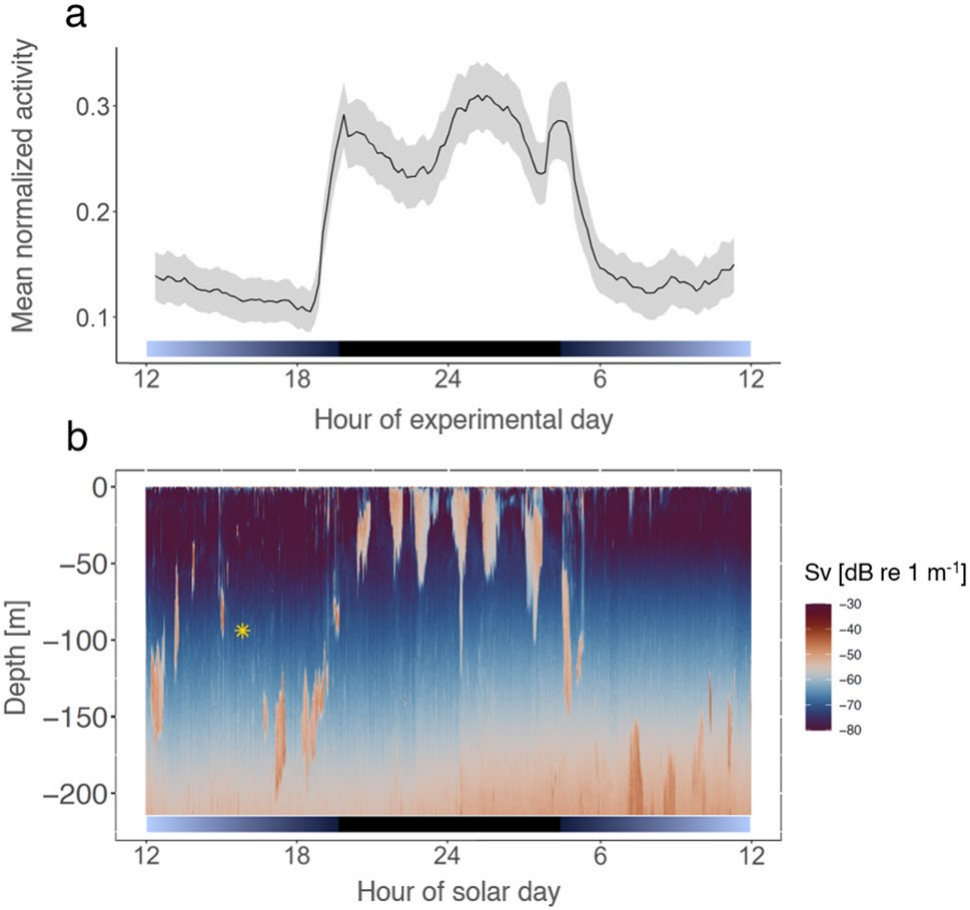
The krill activity profile recorded from the AMAZE set-up reflects krill DVM behavior in the field. Group swimming activity (mean ± s.e.m., n = 9) in the activity monitor visualized over an average experimental day (**a**). Lines with shading depict mean ± s.e.m. Vertical distribution of krill swarms below the vessel visualized by the mean volume backscattering signal of hydroacoustic recordings during a daily cycle following krill sampling (**b**). The asterisk indicates the time and depth of krill sampling for the behavioral experiment. Light bars depict light conditions in the activity monitor (**a**) and local photoperiod (**b**).

This activity metric shows an increase in swimming activity during the dark phase and a decrease during the light phase (see Fig. 4). This pattern is consistent regardless of krill phototactic behavior, as demonstrated by the activity patterns of individuals #4 and #1 (see Fig. 4a-d), despite their differing phototactic behavior (see Fig. 2 and suppl. Figure 3). Consequently, the group activity shows a similar pattern of increased mean activity during the dark phase (Fig. 4e,f). To determine the general daily behavioral activity profile of krill, the data from the group behavior analysis was used to calculate an average day of krill activity over all five experimental days. The daily activity profile demonstrates the nocturnal nature of krill swimming activity and its tight synchronization with the daily light–dark cycle (5a). Similarly, a tight synchronization with the light–dark cycle is apparent in the DVM behavior of krill swarms below the vessel as shown by hydroacoustic recordings taken during the day of sampling (see Fig. 5b), where krill swarms initiate their ascend to the surface, and then descend to deeper layers around the time of sunset and sunrise, respectively.

## Discussion

Here, we introduce AMAZE, a new set-up to record krill swimming behavior under controlled temperature and light conditions. In a first experiment with the AMAZE set-up, conducted onboard a modern krill fishing vessel, we observed individual differences in the behavioral response of wild-caught krill to light–dark cycles. Further, by applying a new swimming activity metric, we could, for the first time, show the nocturnal nature of krill swimming activity in a controlled laboratory setting. The AMAZE set-up thus represents a promising tool for future investigations into aspects of krill behavior, which may contribute to ecologically important behavioral patterns, such as DVM, which have major implications for the structure of the krill-centered ecosystem and related biogeochemical cycles.

Our behavioral recordings showed several individuals attracted by the light source in our activity monitor (Fig. 2, suppl. Figure 2). Attraction to artificial light (i.e. positive phototaxis) is a common phenomenon in marine organisms. It has been shown in previous studies for different krill species (*Thysanoessa inermis*^39^, *Meganyctiphanes norvegica*^40^) including *E. superba*^25,41,42^. The positive phototaxis exhibited in response to artificial light sources contrasts the behavioral patterns observed in nature, where krill commonly move to darker parts of the water column during the day (e.g. DVM,^13,43,44^). In our set-up, the peak in spectral light intensity was slightly shifted to shorter wavelengths (~ 450 nm) compared to field measurements (~ 490 nm), which could have caused the unnatural behavioral response of krill.

While studies on the effects of light spectrum on behavioral responses of krill are scarce, a study on the behavioral attraction and activity of *M. norvegica* found wavelengths within the range of increased spectral sensitivity (i.e. 448 to 505 nm) to be equally attractive. In line with that, a study on the vertical swimming behavior of a group of *E. superba* under light–dark conditions, using light with a peak spectral intensity of 490 nm, found a similar positive phototaxis, with krill higher in a vertical tank during the light phase^25^. The findings have been attributed to a combination of factors, including the artificial conditions of the laboratory environment (e.g. physical constriction, artificial light regimes, and feeding schedules before the start of the experiment). The spectral sensitivity of Antarctic krill covers a range of ~ 400 to 550 nm, with a peak sensitivity at ~ 487 nm^45^, which matches well the natural light spectrum experienced by krill in the field, but also includes sensitivity to a range of wavelengths around this peak. As positive phototactic behavior of *E. superba* seems to be present in studies using diverse light sources and spectra, other factors like the directionality of light might play a more important role in causing the positive phototaxis. In fact, the present study, as well as previous studies showing positive phototaxis in krill^25,41,42^, have in common that they all use directional light sources. While we aimed to reduce this effect by diffusing the light, the confined space in the incubator does not allow to create a perfectly homogenous light field, suggesting that light directionality might add to the factors inducing positive phototaxis in krill.

Our experiment conducted at the individual level revealed that only a portion of the animals displayed a clear positive phototaxis towards the provided light. Further, the animals were not fed during the recordings, suggesting that other internal factors, such as the individual metabolic state during the experiment, could play a role in regulating phototactic behavior, as has been suggested for different zooplankton species^46–48^. However, most studies investigating krill behavioral responses to light stimuli focused on using bright white light to increase the efficiency of krill catches^41,42,49,50^ and systematic investigations of individual behavioral responses to different wavelengths and intensities simulating natural light are missing. Our findings showcase the potential of the AMAZE set-up to systematically study directional behavioral responses to various light conditions, including different wavelengths, intensities, and simulations of their dynamic changes over the course of the day and depth in future experiments.

Studying the mechanistic basis of individual behavior requires establishing experimental conditions that allow the recording of robust phenotypes, which can then be studied under the influence of external stimuli. However, the pelagic lifestyle of krill, with a constant need for swimming and without apparent rest-activity cycles, has complicated the identification of clear behavioral phenotypes in laboratory settings^25,26^. In our study, we have found that separating different aspects of behavior, such as active swimming from passive sinking and hovering, is crucial for measuring a consistent behavioral response under LD cycles. Considering only the upward swimming movements of krill in our set-up allowed us to disentangle phases of baseline activity from increased activity, which revealed the nocturnal character of swimming activity in krill. The activity increase during the dark phase appears highly synchronized with the provided light–dark cycle, similar to the vertical migration patterns of the krill swarms in the field, which the experimental animals were sampled from (Fig. 5). These findings further complement previous observations of increased swimming speed during nighttime for both *E. superba*^51^ and *M. norvegica*^52^ in in-situ studies using hydroacoustic methods. The increased swimming speeds during the night have been related to foraging and feeding behavior, where krill increase the chance of encountering food particles when swimming at higher speeds. In addition, swimming in krill comes with high metabolic costs^38,53^. Reducing swimming activity during the day when hiding from predators at depths would be an effective mechanism to save energy. Other studies have suggested additional energetic benefits of DVM from migrating into deeper, cooler waters during daytime^54–56^, but these studies have largely been restricted to environments with vertical temperature gradients that are much higher compared to the Southern Ocean.

While the new activity metric provides us with the possibility to test a robust phenotype against various external stimuli, it is important to note that limiting the analysis of behavioral phenotypes to single aspects of swimming behavior does not provide a full representation of the complex behavior krill exhibits under natural conditions. Our activity metric focuses on the upwards swimming movements of individual krill. It thus neglects other aspects of swimming behavior, such as swimming speed, horizontal movements or swarming behavior which may be equally important to understand ecologically significant behavioral patterns such as DVM. While decades of research provided a rich basis of observational data^13,16,57^, we are still lacking a mechanistic understanding of how the complex interplay between various internal and external factors drives the krill behavior observed. Reducing the complexity by separating aspects of krill swimming may thus provide a first step to reveal this interplay. Following this approach in future experiments may allow us to focus on other aspects of krill behavior to provide a more complete representation. Combining these experimental insights with the analysis of an increasing resource of hydroacoustic observations may allow to increase our understanding of the ecologically and biogeochemically important behavioral patterns.

The first data from the AMAZE set-up, presented here, suggests that krill swimming activity is a meaningful phenotype. It allows us to study the mechanisms underlying aspects of individual krill swimming behavior and how they might contribute to ecologically essential behaviors such as DVM.

Species of the *Euphausiacea* order are present in all of the world´s oceans^58^. Like Antarctic krill, several pelagic species play a crucial role in their respective ecosystems. They serve as an energy link between primary producers and higher trophic levels^59^, substantially impacting carbon sequestration^60^. However, studying their distribution and abundance is challenging due to the vast spatial dimensions of their habitat and their pelagic lifestyle. Models are essential tools for integrating data from empirical studies to provide estimates and predictions of population dynamics. To refine these models, it is crucial to have a mechanistic understanding of species behavior at the individual level. The set-up is designed to be transportable and capable of simulating temperature and light conditions in krill habitats ranging from the tropics to the poles. Further, exposing animals to reversed or shifted light regimes and various combinations of light spectra and intensities will reveal the influence of light spectrum and intensity on krill swimming behavior. In addition, exposing animals to extended periods of constant conditions (constant light or darkness) will help to disentangle external and internal drivers of krill swimming activity. Extending these experimental studies to other important pelagic krill species will contribute to a more general understanding of the mechanistic principles that underlie krill behavior. By studying the drivers underlying behavioral patterns in different krill species using the AMAZE set-up, we can gain insights that will further help to provide a more general understanding of the mechanisms underlying behavioral patterns in pelagic zooplankton. Incorporating these findings into population models will provide a deeper understanding of how climate change and anthropogenic stressors will affect zooplankton behavior in the future, which has large implications for assessing the functioning of pelagic ecosystems and the related biogeochemical cycles.

### Supplementary Information

Supplementary Figures.

## Data Availability

The datasets and analysis scripts used for the analysis of krill behavior recorded with the AMAZE set-up, as well as hydroacoustic recordings and comparative light measurements are available under https://zenodo.org/records/12792874. Supplementary figures are available in the Supplementary Material file. Technical details of the AMAZE set-up (e.g. technical drawings, detailed list of materials and components, scripts) are available upon request from the corresponding author.
