# Supplementary material for: A new Activity Monitor for Aquatic Zooplankter (AMAZE) allows the recording of swimming activity in wild-caught Antarctic krill (Euphausia superba)
Source: Sci Rep. 2024 Jul 23;14:16963. doi: 10.1038/s41598-024-67999-3 (PMC11266396; doi:10.1038/s41598-024-67999-3)
Supplement: Supplementary file 1 — Supplementary Figures. [file 41598_2024_67999_MOESM1_ESM.docx]

**Supplementary Material to**

“A new Activity Monitor for Aquatic Zooplankter (AMAZE) allows the recording of swimming activity in wild-caught Antarctic krill (*Euphausia superba*)”

**Lukas Hüppe^1,2*^, Dominik Bahlburg^2^, Michael Busack^3^, Johannes Lemburg^4^, Laura Payton^2,5^, Nils Reinhard^1^, Dirk Rieger^1^, Charlotte Helfrich-Förster^1^, Bettina Meyer^2,6,7*^**

**Affiliations:**

**^1^** Neurobiology and Genetics, University of Würzburg, Biocentre, Theodor-Boveri-Institute, Am Hubland, 97074 Würzburg, Germany.

**^2^** Section Polar Biological Oceanography, Alfred-Wegener-Institute for Polar and Marine Research, Am Handelshafen 12, 27570 Bremerhaven, Germany.

**^3^** Section Deep-Sea Ecology and Technology, Alfred-Wegener-Institute for Polar and Marine Research, Am Handelshafen 12, 27570 Bremerhaven, Germany.

**^4^** Scientific Workshop, Alfred-Wegener-Institute for Polar and Marine Research, Am Handelshafen 12, 27570 Bremerhaven, Germany.

**^5^** University of Bordeaux, CNRS, Bordeaux INP, EPOC, UMR 5805, 33120 Arcachon, France.

**^6^** Institute for Chemistry and Biology of the Marine Environment, University of Oldenburg, Carl-von-Ossietzky-Straße 9-11, 26111 Oldenburg, Germany.

**^7^** Helmholtz Institute for Functional Marine Biodiversity at the University of Oldenburg (HIFMB), Ammerländer Heerstrasse 231, 26129 Oldenburg, Germany.

**Contact information:**

lukas.hueppe@uni-wuerzburg.de
bettina.meyer@awi.de

**
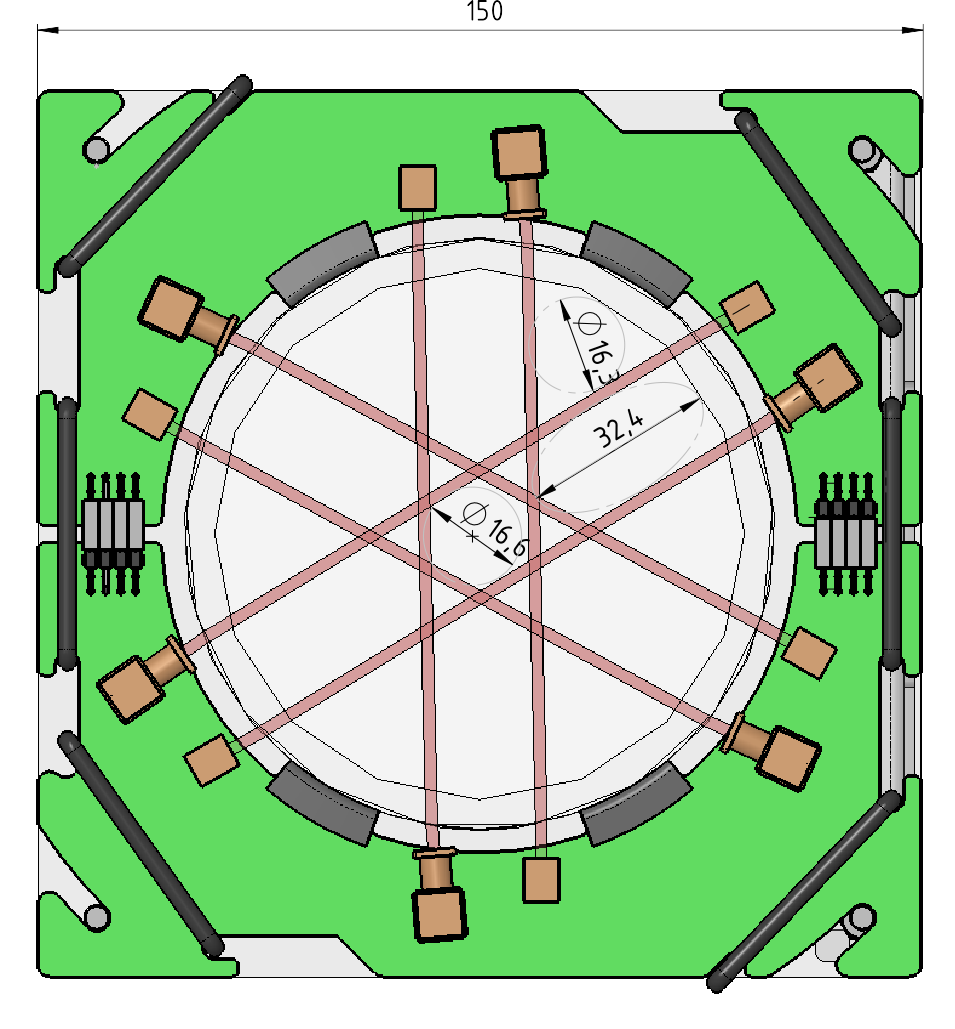
**

**Supplementary Figure 1:**

Technical drawing of a detector module. Dimensions and distances between infrared light beams are given in millimeters.

**
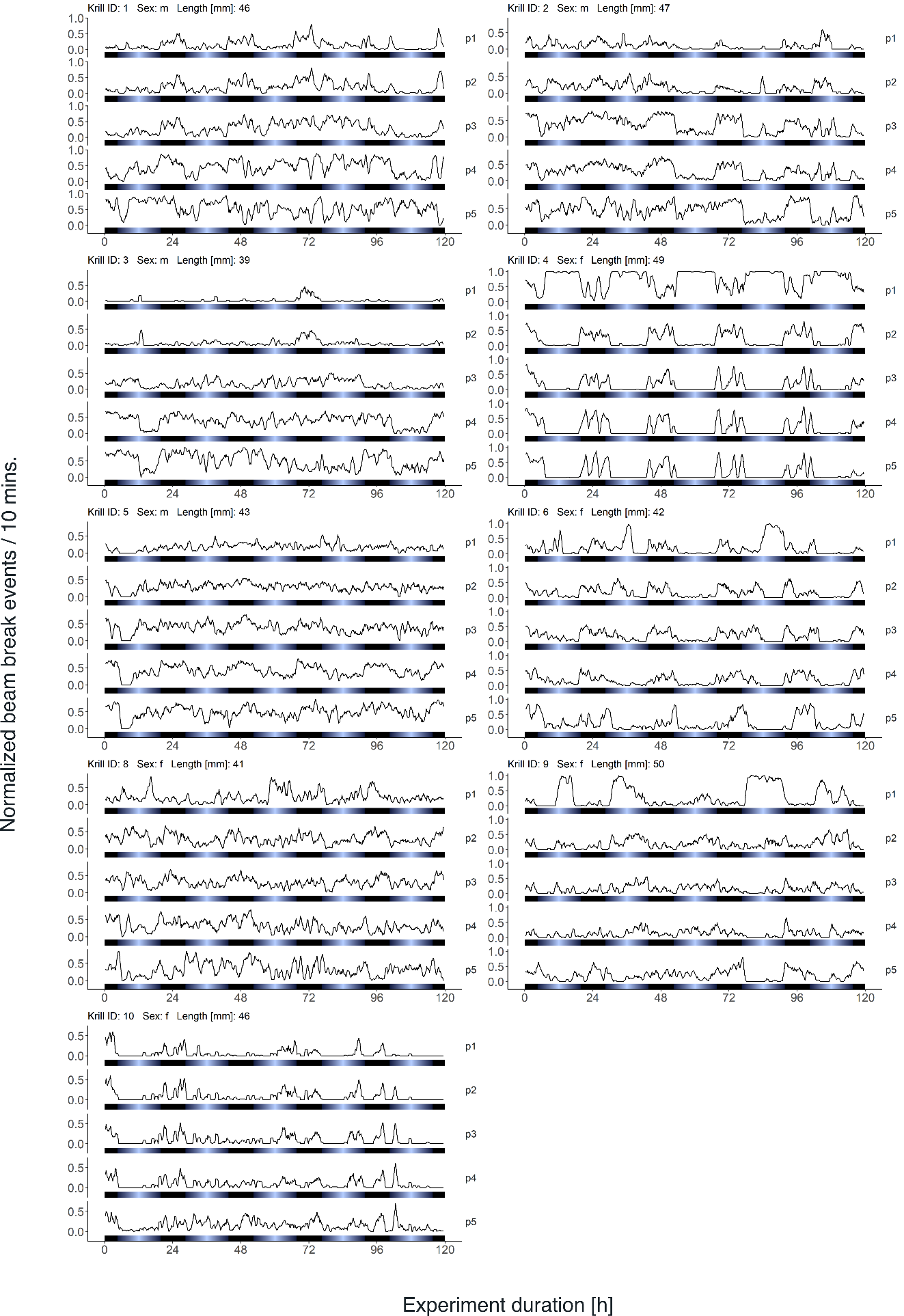
**

**Supplementary Figure 2:**

Normalized beam break events of all krill individuals across the five detector modules (p1-p5) over the duration of the experiment. Color bars at the bottom of each plot depict the light regime provided.

**
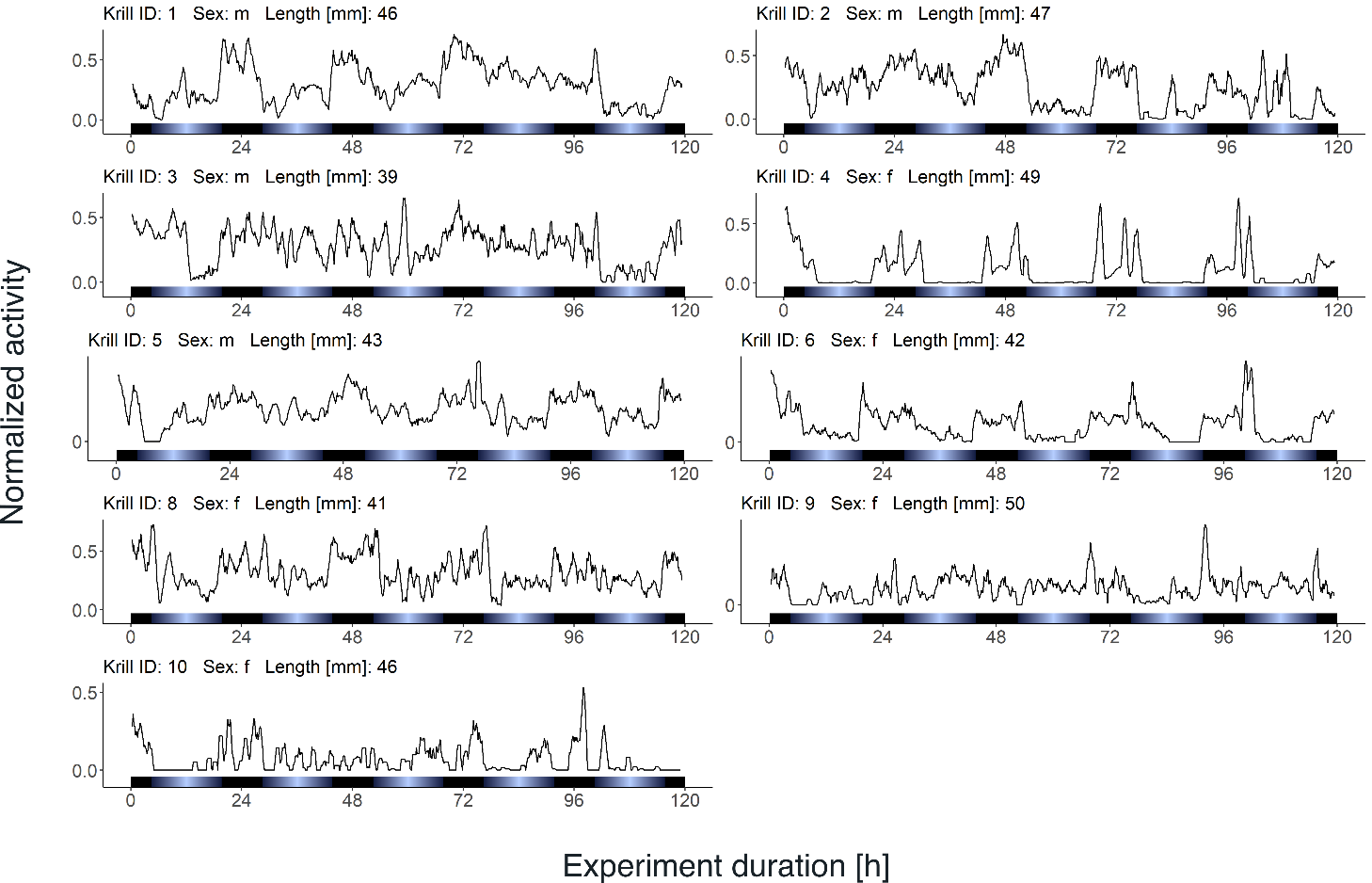
Supplementary Figure 3:**Normalized swimming activity of all krill individuals under light-dark cycles over the duration of the experiment. Color bars at the bottom of each plot depict the light regime provided.
